# Educational Association

**Published:** 1883-06

**Authors:** 


### Educational Association.

In 1866 the faculties of the different dental colleges of this country, formed an association, the object of which was to promote the interest of dental education. The organization was effected with good promise for the future. It continued for two or three years, working harmoniously, and accomplishing something each meeting for dental education. At the third meeting, one college withdrew, without even intimating a reason therefor, then another college in the same city seemed impelled to take the same course, then one in a neighboring city, for self-protection, took the same course. As a result, the association was abandoned, for the time at least. From that to the present, no effort has been made for its revival. There were then six colleges in the United States, now there are about twenty. Certainly, if when there were six, such an association was desirable, now that there are twenty, it is more desirable and important. It has been suggested by the representatives of several of the colleges, that a meeting be held at Niagara, at the time of the meeting of the American Dental Association, with the view of reviving the Association, or of forming a new one. This would certainly be a step in the right direction, and it seems eminently desirable that such a movement be made. Let all interested consider the matter, and in some way give expression to their opinions and conclusions in the matter.

